# Circular RNA SIPA1L1 promotes osteogenesis via regulating the miR-617/Smad3 axis in dental pulp stem cells

**DOI:** 10.1186/s13287-020-01877-3

**Published:** 2020-08-24

**Authors:** Xingyun Ge, Zehan Li, Zhou Zhou, Yibo Xia, Minxia Bian, Jinhua Yu

**Affiliations:** 1 Institute of Stomatology, Nanjing Medical University, 136 Hanzhong Road, Nanjing, 210029 Jiangsu China; 2 Key Laboratory of Oral Diseases of Jiangsu Province and Stomatological Institute of Nanjing Medical University, 140 Hanzhong Road, Nanjing, 210029 Jiangsu China; 3 Peninsula Dental School, Faculty of Medicine and Dentistry, University of Plymouth, Plymouth, UK; 4 Endodontic Department, School of Stomatology, Nanjing Medical University, 136 Hanzhong Road, Nanjing, China

**Keywords:** Circular RNA SIPA1L1, miR-617, Smad3, Dental pulp stem cells, Osteogenesis

## Abstract

**Background:**

Bone regeneration is preferred for bone loss caused by tumors, bone defects, fractures, etc. Recently, mesenchymal stem cells are considered as optimistic tools for bone defect therapy. Dental pulp stem cells (DPSCs) are a promising candidate for regenerative medicine and bone regeneration. Our previous study showed that upregulated circSIPA1L1 during osteogenesis of DPSCs is of significance. In this paper, the potential role of circSIPA1L1 in osteogenesis of DPSCs and its underlying mechanisms are explored.

**Methods:**

The circular structure of circSIPA1L1 was identified by Sanger sequencing and PCR. Regulatory effects of circSIPA1L1 and miR-617 on mineral deposition in DPSCs were assessed by alkaline phosphatase (ALP) and alizarin red S (ARS) staining and in vivo bone formation assay were conducted to verify the biological influences of circSIPA1L1 on DPSCs. Western blot was performed to detect the protein expression of Smad3. Localization of circSIPA1L1 and miR-617 was confirmed by FISH. Dual-luciferase reporter assay and rescue experiments were conducted to investigate the role of the circSIPA1L1/miR-617/Smad3 regulatory axis in osteogenesis of DPSCs.

**Results:**

Sanger sequencing and back-to-back primer experiments confirmed the closed-loop structure of circSIPA1L1. CircSIPA1L1 could promote the committed differentiation of DPSCs. MiR-617 was predicted to be the target binding circSIPA1L1 through MiRDB, miRTarBase, and TargetScan database analyses, which was further confirmed by dual-luciferase reporter assay. FISH results showed that circSIPA1L1 and miR-617 colocalize in the cytoplasm of DPSCs. MiR-617 exerted an inhibitory effect on the osteogenesis of DPSCs. Knockdown of circSIPA1L1 or upregulation of miR-617 downregulated phosphorylated Smad3. In addition, rescue experiments showed that knockdown of miR-617 reversed the inhibitory effect of circSIPA1L1 on osteogenesis of DPSCs.

**Conclusion:**

CircRNASIPA1L1 promotes osteogenesis of DPSCs by adsorbing miR-617 and further targeting Smad3.

## Background

Large bone defects caused by massive injuries, diseases, or deformities can be repaired by autologous bone grafts. Nevertheless, the number of bone grafts is limited and the delicate 3D shape cannot be outlined. Therefore, effective bone regeneration for clinical needs is urgently required. Artificial bone engineering, which creates functional bone tissues using stem cells (the most optimal autologous cells) as an artificial environment or scaffold, contributes to bone defect repair [1, 2]. In recent years, cell therapy, especially mesenchymal stem cells (MSCs), has shown good application prospects in the treatment of bone defects [3]. As an important member of the MSC family, the biological function of bone marrow mesenchymal stem cells has been widely recognized [4]. Due to its strong multi-lineage differentiation potentials and regenerative properties, great progress has been achieved in bone tissue engineering [5]. Sources of stem cells are diverse, including the peripheral blood, bone marrows, cord blood, placenta, and teeth [6]. Notably, dental MSCs are easily available [7].

The endodontium is a special gelatinous soft connective tissue containing the nerves, blood vessels, and connective tissue, which protects the teeth from inflammation and infection [8, 9]. Dental pulp stem cells (DPSCs) are derived from dental pulp tissues, which are featured by high proliferative potential, clonogenicity, self-renewal capacity, and multi-lineage differentiation. As an important mesenchymal stem cell-derived from dental pulp, DPSCs can be collected from the young permanent teeth in a non-invasive way. In addition to the advantages of a wide range of sources and convenient collection, DPSCs also have the advantages of low immunogenicity and no moral controversy [10]. Many studies have shown that DPSCs can differentiate into neurogenic, osteogenic, dentinal, and myogenic cell lineages under different induction conditions [11]. In vivo studies have shown that DPSCs are capable of producing lamellar bones and differentiating into periodontal tissues. Therefore, DPSCs can be utilized for bone regeneration [12]. Moreover, clarifying the osteogenesis of DPSCs is conducive to the development of regenerative medicine and bone disease treatment.

As an important type of ncRNAs, the head-to-tail closed loop structure of circular RNAs (circRNAs) from 3′ end to 5′ tail results in their pronounced stability than traditional linear RNAs [13]. CircRNAs are extensively expressed in thousands of human genes, and sometimes, they exhibit higher expressions than corresponding homologous linear isoforms [14]. CircRNAs mainly exert transcriptional and post-transcriptional regulations on protein sponges [15, 16], translation [17], and miRNA sponges [18]. The well-known competing endogenous RNA (ceRNA) theory of circRNAs has been well concerned. In the nucleotide sequence of circRNA, some circRNAs contain multiple miRNA binding sites capable of binding miRNA, preventing them from binding to their mRNA target genes (sponge effect), thereby inhibiting the function of miRNA. CiRS-7, also known as CDR1as, has been clearly demonstrated as a typical example of miRNA sponge. CircRNA CDR1as contains over 70 miRNA-7 (miR-7) conserved binding sites that strongly inhibit miR-7 activity. After the CDR1as study was published in 2013, various other circRNAs have been shown to act as miRNA sponges. Using bioinformatics tools, miRNA binding sites can be predicted in circRNA sequences. Therefore, for the same miRNA, a circular RNA containing many miRNA binding sites is relatively easy to be found as a miRNA sponge [18–20]. For example, circRNA-ciRS-7 contains over 70 binding sites of miR-7, which are highly conserved and greatly inhibits its activity. However, potential functions of circRNAs in stem cell osteogenesis are rarely reported. We have previously identified differentially expressed circRNAs during osteogenesis of teeth-derived stem cells by RNA sequencing [21], suggesting vital functions of circRNAs in osteogenesis. In the preliminary work, we found that the expression of circSIPA1L1 in the mineralization induction group was about 8 times that of the control group. CircSIPA1L1 is produced by a transcript encoding circSIPA1L1 on human chromosome 14 (NM_015556). Through miRDB, miRTarBase, and TargetScan database analysis, binding sequences in 3′UTR of miR-617 and circSIPA1L1 have been predicted [21]. As is well known, microRNAs (miRNAs) are ncRNAs with short chains (19–25 nt), which inhibiting target gene translation through complementary base pairing [22]. MiRNAs are extensively involved in bone homeostasis through mediating certain cytokines and transcription factors, thereafter affecting the bone formation, remodeling, defect repair, and bone diseases [23]. Dysfunctional miRNAs in osteoporosis have been proven to exert a certain therapeutic potential. Bone destruction and strength are greatly improved in osteoporosis mice intravenously administrated with chemically synthesized miR-106b-5p, miR-17-5p, or miR-451 [24].

This study illustrated the influence of circSIPA1L1 on osteogenesis of DPSCs. Our findings uncovered that knockdown of circSIPA1L1 or overexpression of miR-617 remarkably inhibited osteogenesis of DPSCs. Mechanically, circSIPA1L1 sponged miR-617 to upregulate phosphorylated Smad3. Our results provide potential therapeutic strategies for the bone regeneration through targeting osteogenesis of DPSCs.

## Materials and methods

### DPSC extraction and cell culture

Primarily, the healthy third molars were collected from young people aged 18–25 years after informed consent at Oral and Maxillofacial Surgery of the Jiangsu Provincial Stomatological Hospital. The collection process obeyed the ethical approval of Nanjing Medical University. The tooth was removed and then placed in PBS buffer containing 100 U/mL penicillin. After washing with a sterile saline solution, the surface-adhered gingival tissues and blood clots were removed. Dental pulp was gently harvested in fresh culture medium and digested in 4 mg/mL trypsin (Gibco, Life Technologies, Grand Island, NY) containing 3 mg/mL collagenase type I (Gibco, Life Technologies) at 37 °C. 30 min later, and isolated cells were inoculated in 6-cm culture dishes with α-MEM (Gibco, Life Technologies) containing 10% fetal bovine serum (FBS, Gibco, Life Technologies), 100 μg/mL streptomycin, and 100 U/mL penicillin in a 5% CO_2_ incubator at 37 °C. On the third day, the solution was changed and the medium was replaced every 2 days since after. Cell passage at a ratio of 1:3 was conducted at 70–80% confluence, and third-fifth-generation cells were utilized for subsequent experiments. Isolated DPSCs were induced for osteogenesis at 50–60% confluence in osteogenesis medium (OM, Human Dental Pulp Stem Cell Osteogenic Differentiation Basal Medium, Cyagen Biosciences Inc., USA): standard GM containing 100 μM ascorbic acid, 2 mM 2-glycerophosphate, and 10 nM dexamethasone (Sigma-Aldrich, St. Louis, MO, USA). OM was replaced every 2 days.

### DPSC characterization

STRO-1 is a protein-tagged gene of MSCs, which is the first isolated monoclonal antibody to identify MSCs. Cultured cells (3 days) were subjected to immunofluorescence staining with an antibody of STRO-1 (1:200, Novus Biologicals, Littleton, CO, USA), followed by determination of positive expression of STRO-1. Meanwhile, cells were incubated with CD34-FITC, CD45-PerCP, CD90-PE, CD105-APC, and CD73-PE (Miltenyi, Bergisch Gladbach, Germany) and subjected to FCM analysis (BD Biosciences, CA, USA).

### Tri-lineage differentiation of DPSCs

Mineralized nodule formation of DPSCs was determined by ARS staining, as described previously [25]. Th osteogenesis ability of the third-generation DPSCs was examined by induction in OM for 14 days according to the instructions. Then, DPSCs were reacted in 4% paraformaldehyde for 15 min and dyed with ARS (pH = 4.2, Sigma, Aldrich) for 10 min. ARS was diluted in 10% cetylpyridinium chloride (CPC) to calculate the number of calcified nodules. OD value was determined at 570 nm.

DPSCs were incubated in adipogenic differentiation medium (adult fat adipose-derived stem cell adipogenic differentiation medium, Cyagen Biosciences Inc., USA). When the cell fusion reached 80%, the adipogenic induction group was added with 2 ml OriCell adipogenic differentiation medium A solution. After 3 days, OriCell adipogenic differentiation medium B solution was replaced. After 24 h, change the A solution to culture. After 25 days, Oil Red O staining was conducted to assess adipogenic differentiation in fixed DPSCs.

Three-dimensional pellet culture of DPSCs (2.5 × 10^5^ cells) in a 15-ml sterile tube was conducted for 25-day chondrogenic differentiation. The induction medium was changed every 2 days with the lids of the tube loosened. Pellets fixed and embedded in OCT compounds in a 5-μm thickness (Sakura Finetek Co., Ltd., Tokyo, Japan) were dyed with Alcian Blue.

### Cell transfection

**Fig. 1:**
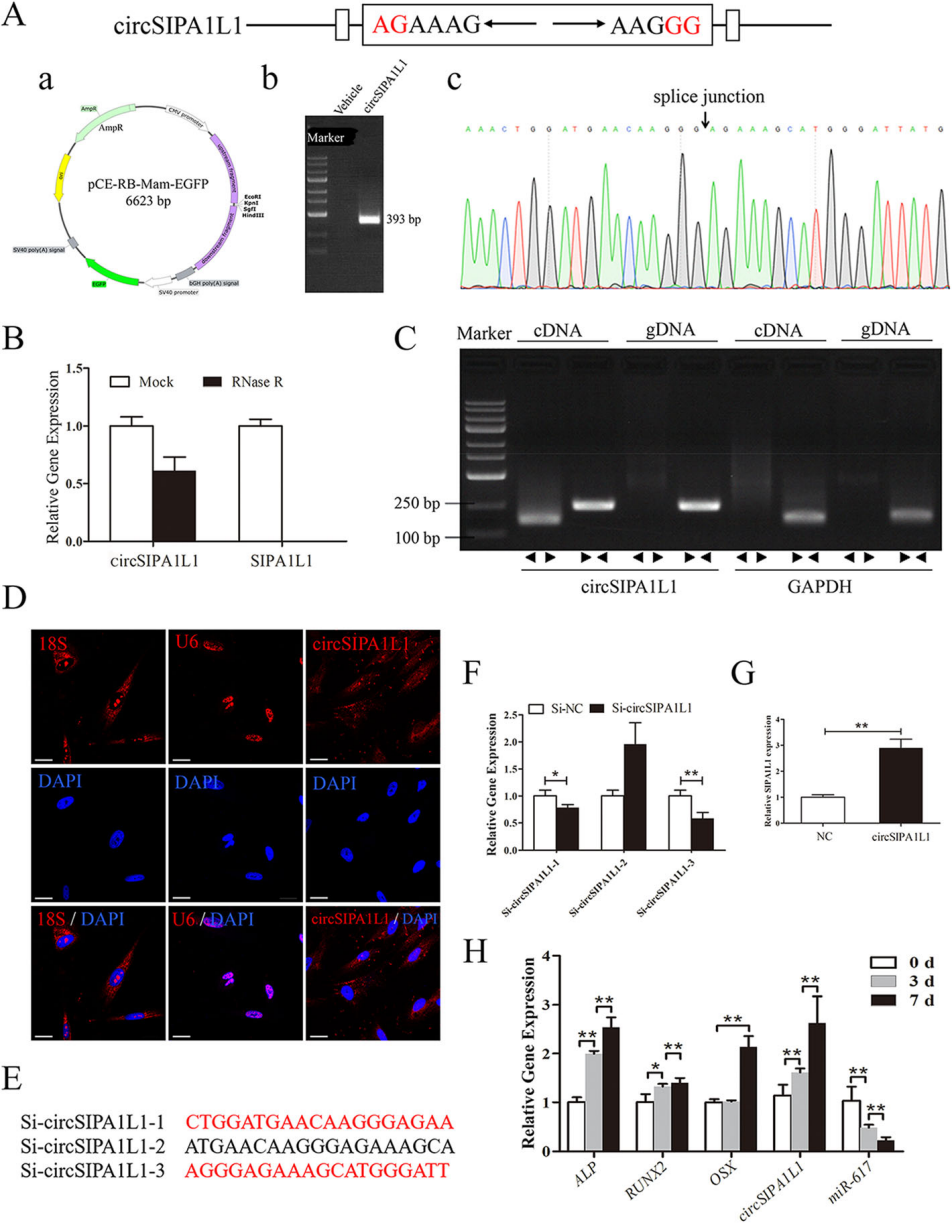
Identification of the circular structure. **a** Head-to-tail splicing of circSIPA1L1 and its genome size and sequences tested by Sanger sequencing. a. Schematic representation of SIPA1L1-expressing plasmid. b. Agarose gel electrophoresis of RT-PCR products from HEK293T cells transfected with SIPA1L1-expressing plasmid or empty vector. c. Sanger sequencing of RT-PCR products from 293T cells, the arrows indicate fusion sites. **b** We further confirmed that circSIPA1L1 was resistant to RNase R. rather than linear-SIPA1L1 could resist digestion by RNase R. **c** The existence of circSIPA1L1 was validated in 293T cell lines by RT-PCR. Divergent primers amplified circSIPA1L1 in cDNA but not genomic DNA (gDNA). GAPDH was used as a negative control. **d** FISH assay showed the localization of circSIPA1L1 in the cytoplasm. 18S and U6 were the internal control. **e** Three circSIPA1L1 small interfering RNAs (siRNAs) specifically targeting the backsplice junction sequences at different binding sites in circSIPA1L1 were designed. **f**. Small interfering RNA silencing efficiency was detected by RT-PCR. The results showed that si-circSIPA1L1-1 and si-circSIPA1L1-3 could effectively knock down the expression of circSIPA1L1(*P < 0.05, **P < 0.01). **g** The expression of circSIPA1L1 between NC and circSIPA1L1 group was detected by RT-PCR. **h** Dynamic expressions of *ALP*, *RUNX2*, and *OSX* during DPSC osteogenesis at days 0, 3, and 7. Dynamic expressions of circSIPA1L1 and miR-617 during DPSC osteogenesis at days 0, 3, and 7. RNA level was normalized to that at day 0. GAPDH and U6 were the internal controls, respectively. *P < 0.05, **P < 0.01

Three circSIPA1L1 siRNAs (100 nM) were designed by Ribobio (Ribobio, China), and their sequences were as follows: siRNA-1: CTGGATGAACAAGGGAGAA; siRNA-2: ATGAACAAGGGAGAAAGCA; siRNA-3: AGGGAGAAAGCATGGGATT (Fig. 1e), the si-NC group was transfected with a randomized sequence of siRNA, the transfection efficacy was tested by RT-PCR, and at last, circSIPA1L1 siRNA-1 and siRNA-3 were selected (Fig. 1f). Meanwhile, miR-617 mimic (50 nM), miR-617 mimic NC (50 nM), miR-617 inhibitor (100 nM), and miR-617 inhibitor NC (100 nM) were purchased from Ribobio as well. To overexpress the circSIPA1L1, we designed an overexpression plasmid of circSIPA1L1 and the NC group was transfected with an empty vector, after transfection using lipofectamine 2000 (Invitrogen, USA) for 48–96 h, the transfection efficacy was tested by RT-PCR complete medium was replaced at 6 h.

### Flow cytometry

DPSCs were cultured for 3 days, then collected by trypsin (Beyotime, Haimen, China), and fixed in alcohol overnight at 4 °C in the dark. After PBS wash, the samples were subjected to FACScan flow cytometer (BD Biosciences, San Jose, CA) and independently analyzed three times.

### Cell proliferation assay

The proliferative potential of DPSCs was determined by the Cell Counting Kit-8 (CCK-8) (Dojindo, Tokyo, Japan) assay and EdU incorporation assay. For CCK-8 assay, 3 × 10^3^ DPSCs were inoculated in each well of a 96-well plate. After 24 h of culture, the medium was replaced with osteogenesis medium. After 1, 3, 5, 7, and 9 days of culture, DPSCs were treated with CCK-8 regents at 37 °C for 2 h and the optical density (OD) at 450 nm was measured by a microplate reader.

For EdU incorporation assay, 5 × 10^3^ DPSCs were treated with 50 mM 5 ethynyl-20-deoxyuridine (EdU, Ribobio) at 37 °C for 6 h. After a 30-min fixation in 4% paraformaldehyde (PFA), DPSCs were treated with 2 mg/ml glycine for 10 min, 0.5% Triton X-100 and 1 × Apollo reaction mixture for 30 min. Subsequently, DPSCs were treated with 1 × Hoechst-33342 solution in the dark for 30 min at room temperature (RT) and images were captured by fluorescence microscopy.

### ALP staining

At day 7 of osteogenesis, DPSCs were fixed in 4% PFA for 15 min and washed with PBS for three times. ALP staining was performed using the NBT/BCIP staining kit (Beyotime, China), and images were captured using a microscope (Olympus, Japan).

### Western blot

RIPA lysis buffer (Beyotime, China) was used to isolate cellular protein, which was further loaded onto 10% SDS-PAGE and transferred to a polyvinylidene fluoride membrane (Millipore, MA, USA). After a 2-h blockage in 5% skim milk, the membrane was incubated with diluted OSX, RUNX2, ALP (Abcam, UK), Smad3, and GAPDH (Cell Signaling Technology) at 1:1000 overnight at 4 °C. After TBST wash for three times, the membrane was reacted with the corresponding secondary antibody for 1 h at RT. The gray value was analyzed by ImageJ software.

### Reverse transcription polymerase chain reaction (RT-PCR)

RNAs extracted from DPSCs using TRIzol (Invitrogen, USA) underwent reverse transcription by PrimeScript RT kit (TaKaRa, Otsu, Japan). RT-PCR was performed on an ABI 7300 real-time PCR system with Universal ChamQTM SYBR Green quantitative PCR Master Mix (Vazyme, Nanjing, China). GAPDH and U6 were the internal references for mRNA and miRNA, respectively. Bulge-Loop miRNA qPCR Primer kit (RiboBio) was used for measuring miRNA-617 expression. Primer sequences for ALP, OSX, RUNX2, and GAPDH were depicted in Table 1. Expression levels were calculated by the 2^−ΔΔCt^ method as previously reported [25].

**Table 1:** Sense and antisense primers for real-time reverse transcription polymerase chain reaction

Genes	Primers	Sequences (5′-3′)
*RUNX2*	Forward	TCTTAGAACAAATTCTGCCCTTT
	Reverse	TGCTTTGGTCTTGAAATCACA
*OSX*	Forward	CCTCCTCAGCTCACCTTCTC
	Reverse	GTTGGGAGCCCAAATAGAAA
*ALP*	Forward	ACCTGAGTGCCAGAGTGA
	Reverse	CTTCCTCCTTGTTGGGTT
*GAPDH*	Forward	GAAGGTGAAGGTCGGAGTC
	Reverse	GAGATGGTGATGGGATTTC

### Immunofluorescence staining

After PBS washing for three times, DPSCs were subjected to a 30-min incubation in 4% paraformaldehyde, a 15-min incubation in 0.1% Triton X-100 (Beyotime), and a 2-h blockage in normal goat serum (DCS/BioGenex, Hamburg, Germany) at RT. After treatment with primary and T fluorescent dye-labeled designated secondary antibody at appointed time points, nuclei were counterstained with DAPI (Beyotime). Immunofluorescence images were observed under a fluorescent inverted microscope (Olympus, Shanghai, China).

### Dual-luciferase reporter assay

Dual-luciferase reporter assay was conducted as described previously [25]. HEK-293 T cells were obtained from the group of Hongbing Jiang from Key Laboratory of Oral Diseases of Jiangsu Province and were cultured in Dulbecco’s Modified Eagle Medium (Gibco, Life Technologies) supplemented with 10% FBS. In brief, HEK293 T cells seeded in 24-well plates (5 × 10^5^ cells/well) were co-transfected with Firefly luciferase reporter vector (800 ng), Renilla luciferase reporter vector (5 ng wild-type or mutant-type, GeneChem, Shanghai, China), and 50 nM miR-617 mimics or negative control using lipofectamine 2000. Luciferase activity measured by the Dual-Luciferase Reporter Assay System (Promega) was finally calculated as Firefly luciferase activity normalized to that of Renilla.

### Animal procedures

Animal procedures followed institutional guidelines and got the approval of the Ethics Committee of Nanjing Medical University. Fifteen 5-week homozygous nude mice were provided by the Animal Center of Nanjing Medical University. Mice were habituated for 1 week with 3–4 per cage. They were randomly assigned into three groups (*n* = 5 per group) and subcutaneously transplanted with DPSCs transfected with si-NC, si-circSIPA1L1-1, or si-circSIPA1L1-3, respectively. Specifically, transfected DPSCs underwent osteogenesis for 2 weeks, followed by treatment with Bio-Oss collagen (Geistlich, Germany) scaffold for 12 h at 37 °C. Make two longitudinal incisions on the back of the nude mouse and bluntly separate to form a dorsal subcutaneous pocket, where two implants were inserted. Eight weeks later, the implant was removed and fixed in 4% PFA.

### Histology

Sections were decalcified in 10% EDTA (pH 7.4) for 4 weeks with EDTA solution replacement every other day, dehydrated, and paraffin-embedded. Subsequently, sections were sagittally sectioned, deparaffinized, and visualized by hematoxylin and eosin (H&E) or Masson’s trichrome staining. Images were captured using a microscope.

### Statistical processing

Statistical Package for Social Sciences (SPSS) software 16.0 was used for statistical analyses. One-way analysis of variance (ANOVA) and Student’s *t* test was used for comparing differences. A two-tailed *P* < 0.05 considered as statistically significant. Data were expressed as mean ± SD of from at least three independent experiments.

## Results

### Phenotype identification of DPSCs

The morphology of primary generation DPSCs were fibroblast- or spindle-like (Fig. S1A). To identify the phenotype and qualification of extracted DPSCs, the multipotency, including chondrogenic, adipogenic, and osteogenic differentiation, was tested [26]. Flow cytometry results demonstrated that the isolated DPSCs were negative for hematopoietic markers (CD34, CD45) (Fig. S1B), but positive for MSC markers (CD29, CD90, CD73, and CD105) (Fig. S1C). Tri-lineage differentiation of DPSCs was firstly confirmed (Fig. S1D). Meanwhile, immunofluorescence staining results showed that DPSCs were positive for the MSC surface molecule STRO-1 (Fig. S1D). The above results all verified the stem cell characteristics of isolated DPSCs.

### Identification of the circular structure

To identify the circular structure of circSIPA1L1, the SIPA1L1-expressing plasmid was designed (Fig. 1a). Head-to-tail splicing of circSIPA1L1 was done, and its genome size and sequences were confirmed by Sanger sequencing (Fig. 1a). Moreover, divergent and convergent primers were used and it is found that circSIPA1L1, but not linear SIPA1L1 was resistant to RNase R digestion (Fig. 1b). To exclude the possibility that head-to-tail splicing product of circSIPA1L1 comes from genomic rearrangement or trans-splicing, its cDNA and gDNA of 293T cells either with RNase R or not were detected. The supplemental expression level of reverse splicing or canonical form of SIPA1L1 was shown (Fig. 1c). Subsequently, FISH identified that circSIPA1L1 was mainly distributed in the cytoplasm of DPSCs with 18S and U6 as the internal control (Fig. 1d). We hypothesized that circSIPA1L1 regulates the biological characteristics of DPSCs via the ceRNA mechanism. In summary, circSIPA1L1 was identified as a stable circRNA and deserved further exploration.

### CircSIPA1L1 is upregulated and miR-617 is downregulated during osteogenesis of DPSCs

Dynamically expressed circSIPA1L1 and miR-617 during osteogenesis in DPSCs were detected. Three circSIPA1L1 small interfering RNAs (siRNAs) specifically targeting the backsplice junction sequences at different binding sites in circSIPA1L1 were designed (Fig. 1e). Small interfering RNA transfection efficiency was detected by RT-PCR. The results showed that si-circSIPA1L1-1 and si-circSIPA1L1-3 could effectively knockdown the expression of circSIPA1L1(Fig. 1f). Meanwhile, the expression of circSIPA1L1 between NC and circSIPA1L1 group were detected by RT-PCR, and the results showed that circSIPA1L1 could effectively increase the expression of circSIPA1L1(Fig. 1g).

CircSIPA1L1 was time-dependently upregulated, and miR-617 was downregulated in osteogenic DPSCs. Moreover, mRNA levels of osteogenesis markers *ALP*, *OSX*, and *RUNX2* were remarkably upregulated during the process of osteogenesis (Fig. 1H), demonstrating the successful induction of osteogenesis.

### CircSIPA1L1 have no effect on DPSC proliferation

To elucidate the role of circSIPA1L1 in DPSC proliferation, CCK-8, flow cytometry, and EdU assay were conducted. FCM analysis did not show significant differences in the proliferation index (PI = G2M ± S) between the NC group (9.15%) and the circSIPA1L1 group (8.15%, *P* > 0.05, Fig. S2A). Similarly, no significant difference was found in the proliferation index between the si-NC group (4.72%), the si-circ-SIPA1L1-1 group (5.23%), and the si-circ-SIPA1L1-3 group (5.32%, *P* > 0.05, Fig. S2A). In addition, the results of the EdU assay showed no significant difference between the NC group and the circSIPA1L1 group (*P > 0.05*, Fig. S2B, C) or between the si-NC, si-circ-SIPA1L1-1, and si-circ-SIPA1L1-3 groups (Fig. S2B, D). The CCK-8 assay showed no significant difference in proliferation rates between the NC group and the circSIPA1L1 group (Fig. S2E) or between the si-NC, si-circ-SIPA1L1-1, and si-circ-SIPA1L1-3 groups from 0 days to 9 days (*P* > 0.05) (Fig. S2F). Taken together, the data demonstrated that circSIPA1L1 does not affect the proliferation of DPSCs.

### CircSIPA1L1 stimulates DPSC osteogenesis

**Fig. 2:**
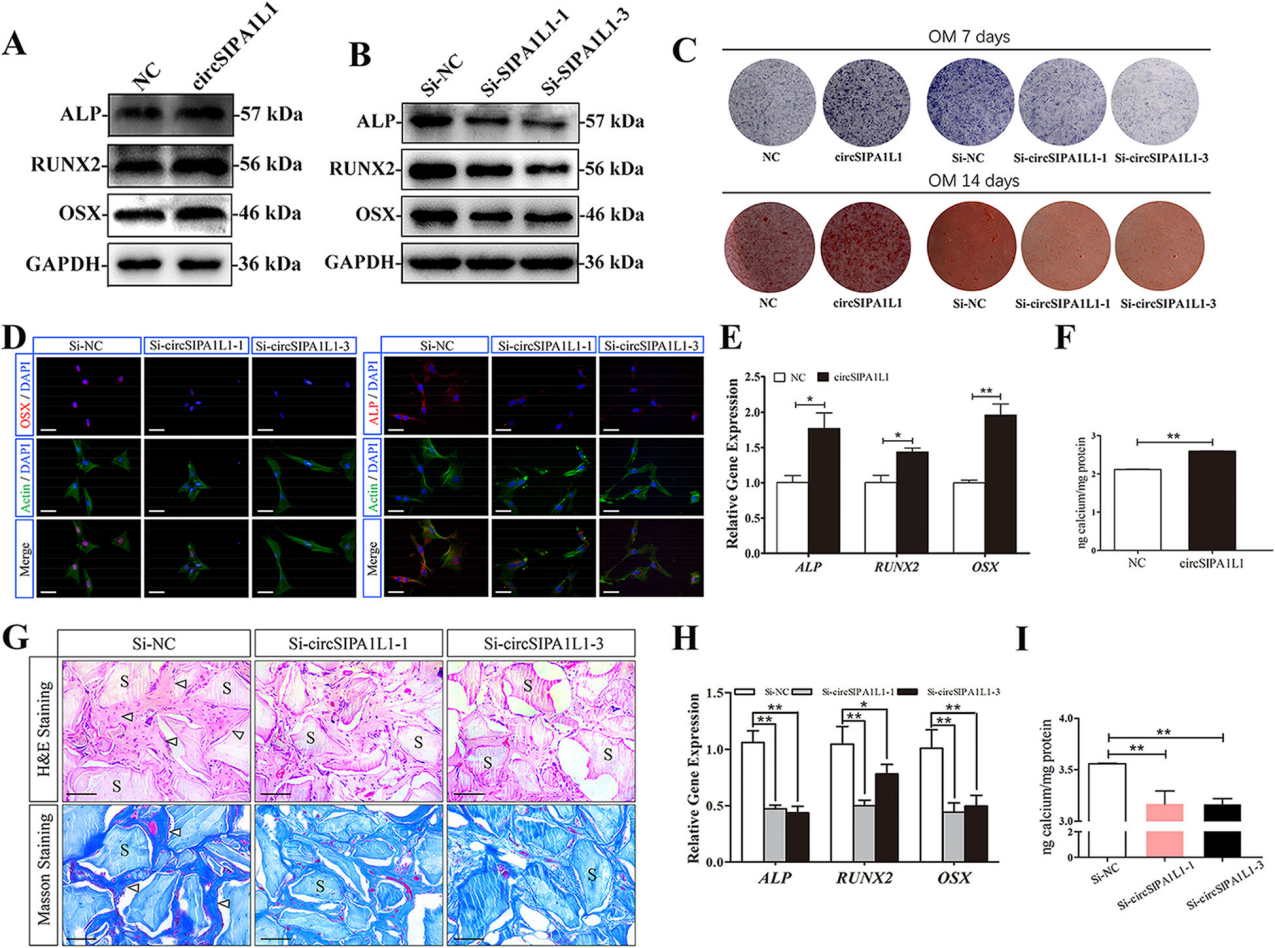
CircSIPA1L1 stimulates DPSC osteogenesis. **a** Western blot results revealed that the protein levels of OSX, RUNX2, and ALP significantly increased in the circSIPA1L1 group compared with the NC group. **b** Western blot assay showed higher protein levels of ALP, RUNX2, and OSX in the Si-NC group than the Si-circSIPA1L1-3 group and Si-circSIPA1L1-1 group, respectively. GAPDH was the internal control. **c** Images of alkaline phosphatase (ALP) staining in the different groups, and Si-NC treatment led to the highest ALP activity. Cells were cultured for 7 days. After 14 days of co-culture, the formation of mineralized nodules in DPSCs in the circSIPA1L1 group generated more calcified nodules than the NC group; meanwhile, the Si-NC group has more calcified nodules than Si-circSIPA1L1-3 group and Si-circSIPA1L1-1 group. **d** Immunofluorescence staining showed positive expressions of ALP and OSX in DPSCs transfected with si-NC, si-circSIPA1L1-1 or si-circSIPA1L1-3, respectively. *P < 0.05, **P < 0.01. **e** The mRNA levels of *OSX*, *RUNX2*, and *ALP* in DPSCs measured by RT-PCR following the 3-day osteogenesis. The results showed higher levels of *ALP*, *RUNX2*, and *OSX* in the circSIPA1L1 group than the NC group. **f** Histograms showed quantification of Alizarin red staining by spectrophotometry of the NC group and circSIPA1L1 group. **g** H&E staining and Masson staining Si-NC, si-circSIPA1L1-1, and si-circSIPA1L1-3 groups. H&E and Masson staining showed less bone-like structures and collagen deposits in DPSCs of the circSIPA1L1-downexpressing group than the control group. Bone/dentin-like tissues (arrow), S around the scaffold, scale bar = 100 μm. **h** The results of RT-PCR showed that the expression of *ALP*, *RUNX2*, and *OSX* were decreased in the Si-circSIPA1L1-3 group and Si-circSIPA1L1-1 group compared with the Si-NC group. *n* = 3. **2^*-ΔΔCt*^ *> 2*, *P < 0.01*; *1 < 2^*-ΔΔCt*^ *< 2*, *P < 0.05.* **i** The quantification of Alizarin red staining by spectrophotometry revealed that si-circSIPA1L1-1 or si-circSIPA1L1-3 showed less calcified nodules than si-NC group

To further analyze the effect of circSIPA1L1 on osteogenic differentiation of DPSCs, protein and mRNA levels of ALP, OSX, and RUNX2 were detected by the Western blot and RT-PCR in osteogenic DPSCs. Western blot results showed that protein expression of ALP, OSX, and RUNX2 were upregulated in the overexpression group of circSIPA1L1 (Fig. 2a). The results of RT-PCR indicated that circSIPA1L1 overexpression increased *ALP*, *OSX*, and *RUNX2* (Fig. 2e), whereas the expression of protein level was downregulated when circSIPA1L1 was knocked down in DPSCs (Fig. 2b), and the results of RT-PCR indicated that the level of *ALP*, *OSX*, and *RUNX2* were decreased in circSIPA1L1 knockdown of DPSCs (Fig. 2h). After 7 days of osteogenesis, ALP staining showed decreased ALP activity after knockdown of circSIPA1L1 and obviously upregulated by circSIPA1L1 overexpression (Fig. 2c). After 14 days of induction, alizarin red staining showed reduced matrix mineralization in DPSCs with circSIPA1L1 knockdown whereas circSIPA1L1 overexpression obtained the opposite effects (Fig. 2c, f, i). Identically, positive expressions of ALP and OSX were downregulated by circSIPA1L1 knockdown in DPSCs as immunofluorescence revealed (Fig. 2d). These results indicated that circSIPA1L1-stimulated DPSC osteogenesis.

DPSCs stably downexpressing circSIPA1L1 and controls were loaded on Bio-Oss Collagen scaffolds and implanted in the subcutaneous tissues of nude mice for 8 weeks of growth. Both H&E and Masson staining showed less bone-like structures and collagen deposits in DPSCs of the circSIPA1L1-downexpressing group than the control group (Fig. 2g).

### CircSIPA1L1 sponges miR-617

**Fig. 3:**
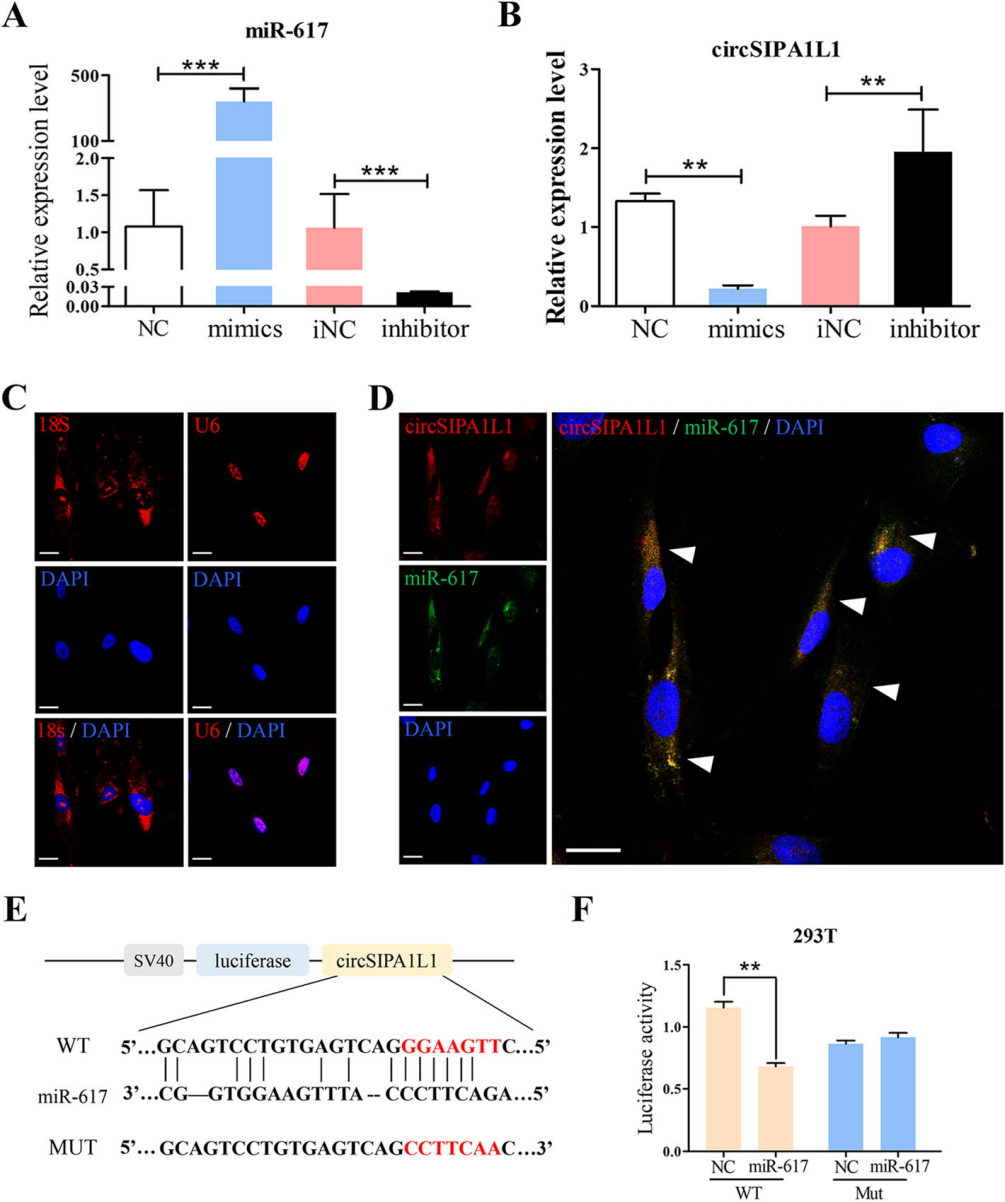
CircSIPA1L1 sponges miR-617. **a** RT-PCR assay showed the transfection efficacy of NC, miR-617 mimics, miR-617 inhibitor, and inhibitor NC group. **b** Relative circSIPA1L1 expression level in DPSCs transfected with miR-617 mimics or inhibitor (**P < 0.01). **c**, **d** FISH showed co-localization of circSIPA1L1 and miR-617 in the cytoplasm of DPSCs cells (arrow). U6 and 18S were the internal control. **e** The potential binding sequences in 3′UTR of circSIPA1L1 and miR-617 predicted online. **f** Luciferase activity in 293T cells co-transfected with miR-617 mimics/NC and wild-type/mutant-type circSIPA1L1, respectively. *P < 0.05, **P < 0.01

CircRNAs are able to regulate downstream gene expressions and functions by sponging corresponding miRNAs. The transfection efficiency of miR-617 mimics, and inhibitor was verified by RT-PCR (Fig. 3a). It is shown that circSIPA1L1 expression was negatively regulated by miR-617 (Fig. 3b). To further validate their interaction, the FISH analysis was conducted in DPSCs, and the results revealed that miR-617 colocalized with circSIPA1L1 in the cytoplasm (Fig. 3c, d). Through analyses on miRDB, miRTarBase, and TargetScan database, a binding site in 3′UTR of miR-617 and circSIPA1L1 was discovered (Fig. 3e). Subsequently, dual-luciferase reporter assay was conducted to test the interaction between circSIPA1L1 and miR-617. 293T cells were co-transfected with miR-617 mimics/negative control and wild-type/mutant-type circSIPA1L1, respectively. Overexpression of miR-617 markedly quenched luciferase activity in wild-type circSIPA1L1 compared with controls, verifying the direction interaction between circSIPA1L1 and miR-617 (Fig. 3f). These observations indicated that circSIPA1L1 and miR-617 coexisted in the cytoplasm, and circSIPA1L1 acted as a miRNA sponge for miR-617 in DPSCs.

### MiR-617 inhibits DPSC osteogenesis

**Fig. 4:**
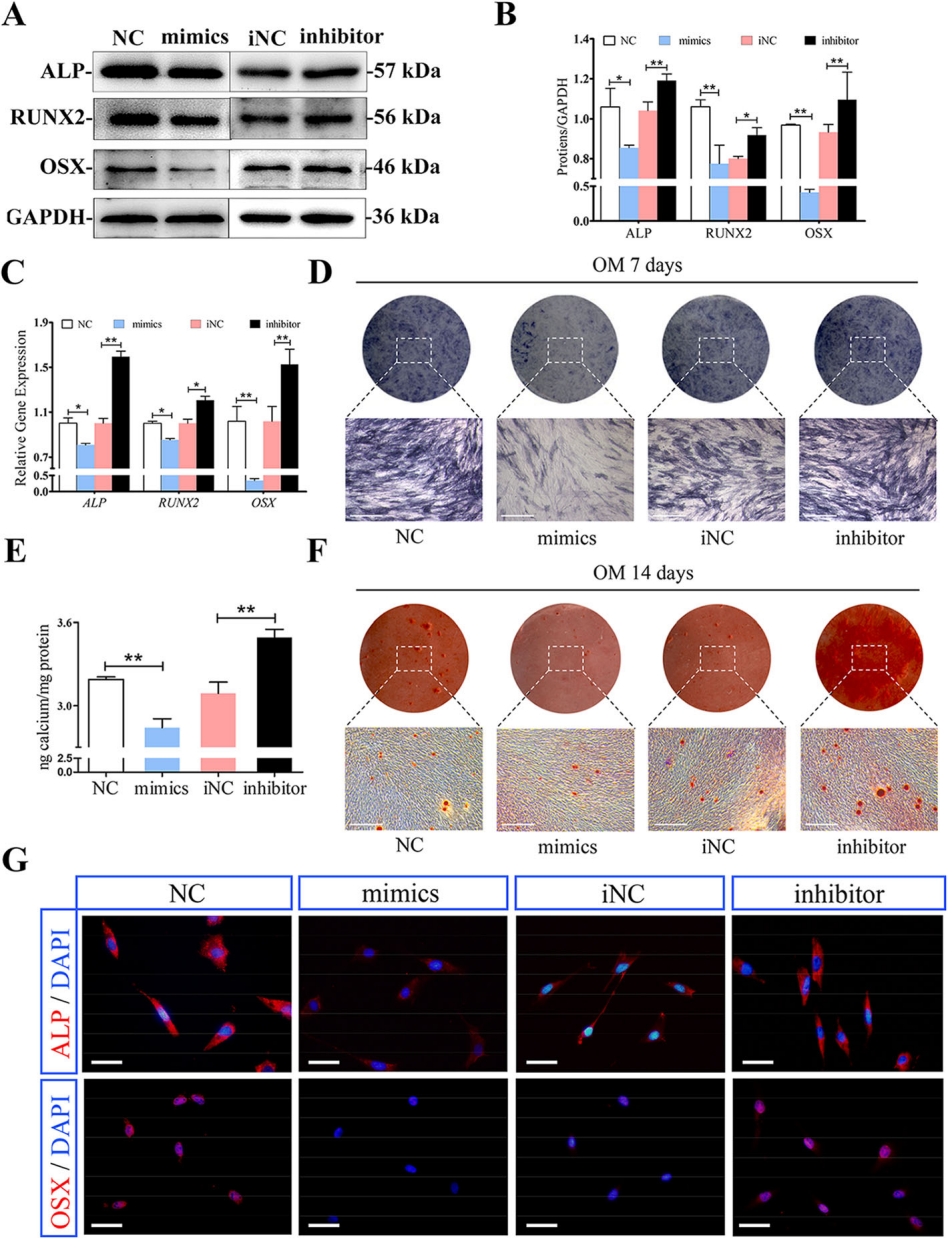
MiR-617 inhibits DPSCs osteogenesis. **a** Western blot assay showed higher protein levels of ALP, RUNX2, and OSX in the NC group and miR-617 inhibitor group than mimics group and miR-617 inhibitor NC group respectively. GAPDH was the internal control. **b** Grayscale analyses. *P < 0.05 or **P < 0.01. **c** RT-PCR showed higher levels of *ALP*, *RUNX2*, and *OSX* in the NC group and inhibitor than mimics group and iNC group, respectively. **d** ALP staining in DPSCs following a 7-day osteogenesis with overexpression or knockdown of miR-617. **e** Histograms showed quantification of Alizarin red staining by spectrophotometry. **f** After 14 days of co-culture, upper: alizarin red staining showed that miR-617 mimics group generated more calcified nodules than the control group. MiR-617 inhibitor group generated more calcified nodules than the iNC group. Lower: mineralized nodules in different groups under the inverted microscope (OM: osteogenic medium). **g** Immunofluorescence showed positive expressions of ALP and OSX in DPSCs with overexpression or knockdown of miR-617. *P < 0.05, **P < 0.01

Next, the potential influence of miR-617 on DPSC osteogenesis was explored. Western blot revealed that the protein levels of ALP, OSX, and RUNX2 increased in the miR-617 knockdown group and the opposite effect was observed in the miR-617 overexpression group (Fig. 4a, b). RT-PCR analysis confirmed that the expression of osteogenic related genes *ALP*, *OSX*, and *RUNX2* were significantly lower in the miR-617 overexpressing group than in the control group, while knockdown of miR-617 increased the gene expression of these osteogenic markers (Fig. 4c). After 7 days of osteogenesis, ALP staining showed that miR-617 negatively regulated ALP activity in DPSCs (Fig. 4d). After 14 days of osteogenesis, alizarin red staining showed that the formation of mineralized nodules in DPSCs was negatively mediated by miR-617 as well (Fig. 4e, f). Immunofluorescence staining analysis showed that positive expressions of ALP and OSX were upregulated in DPSCs with the miR-617 knockdown group, which were downregulated in those overexpressing miR-617 group (Fig. 4g). In conclusion, the above findings demonstrated that miR-617 was a negative regulator in DPSC osteogenesis.

### MiR-617 directly targets Smad3

**Fig. 5:**
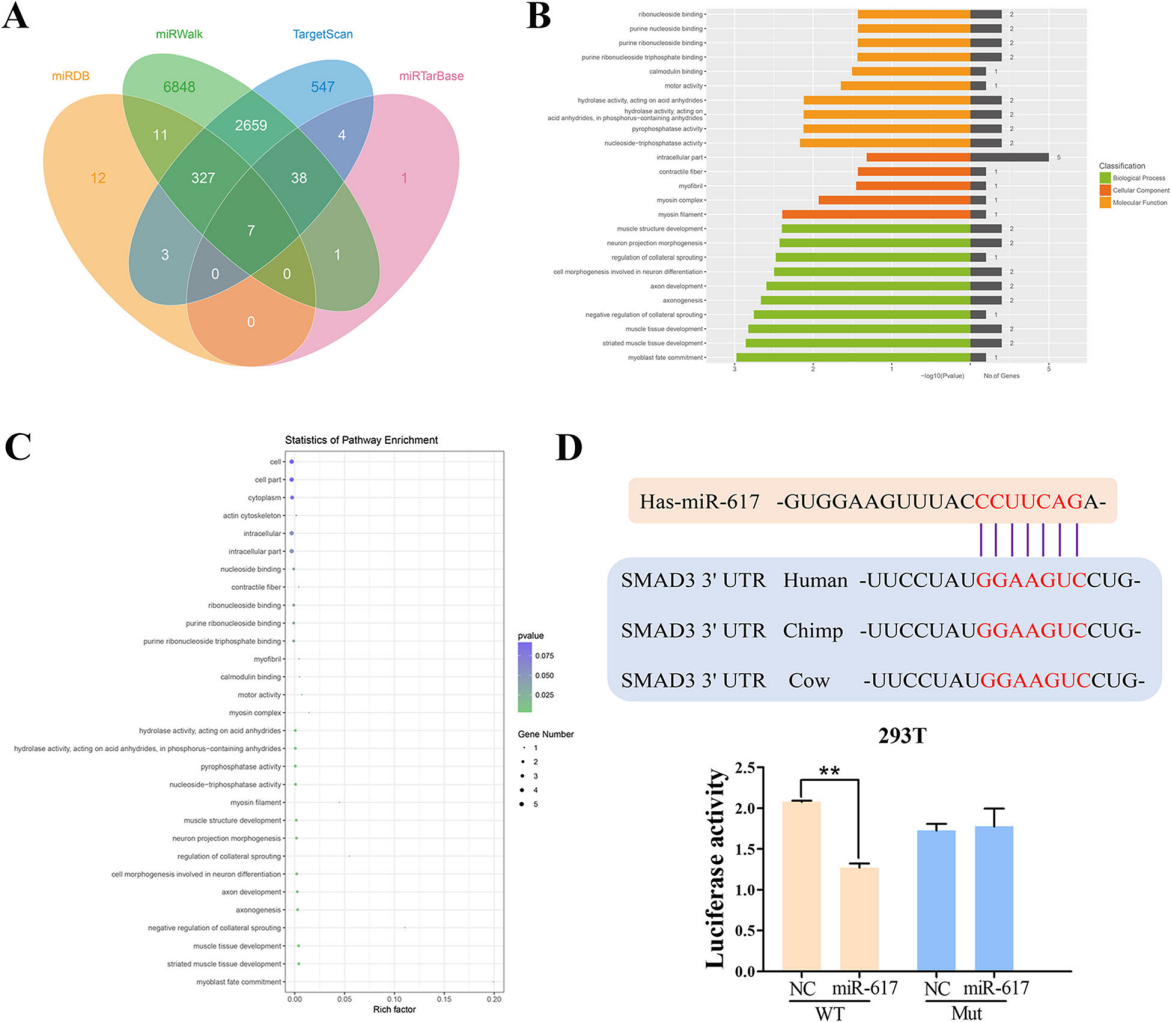
MiR-617 directly targets Smad3. **a** The Venn plots showed predicted downstream genes binding miR-617. **b**, **c** Go annotation (**b**) and KEGG pathway analysis (**c**) showed the top 25 target genes and their enriched pathways. GO: A field directly related to reproduction. **d** Binding sequences in 3′UTR of miR-617 and Smad3 predicted online. The red letters represent the binding sequence of miR-617. Luciferase activity in 293T cells co-transfected with miR-617 mimics/NC and wild-type/mutant-type smad3, respectively. GO: Gene ontology; KEGG: Kyoto Encyclopedia of Genes and Genomes

Similarly, downstream genes binding miR-617 were predicted using the miRDB, miRTarBase, miRWalk, and TargetScan algorithms (Fig. 5a). A total of 10,461 potential target genes of miR-617 were obtained (see supplement file 1). GO and KEGG pathway analysis indicated that these target genes were mainly involved in intracellular activities (Fig. 5b, c). Interestingly, Smad3 was a shared gene predicted in the miRDB, miRWalk, and TargetScan databases. As an intracellular protein, Smad3 induces nuclear transportation of extracellular transforming growth factor β ligands, thereafter activating transcription of downstream genes. Binding sequences in 3′UTR of Smad3 and miR-617 were shown (Fig. 5d), and the complementary regions between these different species were also highly conserved.

### CircSIPA1L1/miR-617/Smad3 axis is responsible for DPSC osteogenesis

**Fig. 6:**
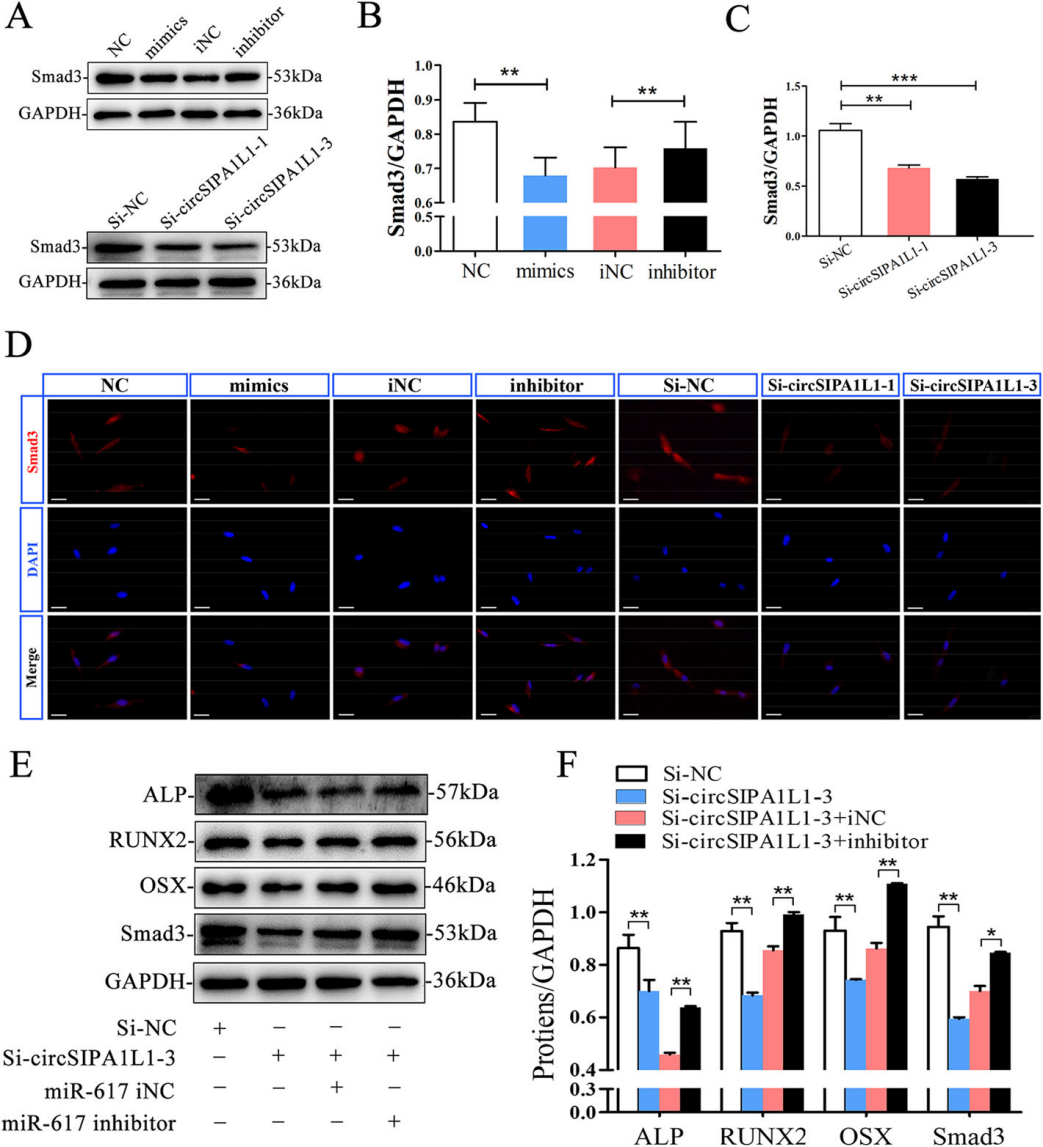
CircSIPA1L1/miR-617/Smad3 axis is responsible for DPSCs osteogenesis. **a** Western blot assay showed higher protein levels of Smad3 in the miR-NC group and miR-617 inhibitor than the miR-617 mimic group and miR-617 inhibitor NC group respectively. Meanwhile, the si-circSIPA1L1-downexpressing group showed lower protein levels of Smad3 than the control group. **b**, **c** Grayscale analyses. **P < 0.01 or ***P < 0.001. **c** Immunofluorescence assay revealed upregulated Smad3 in the NC group and miR-617 inhibitor than miR-617 mimics group and miR-617 inhibitor NC group, respectively. **d** Immunofluorescence assay revealed upregulated Smad3 in the Si-NC group compared with Si-circSIPA1L1-3 group and Si-circSIPA1L1-1 group. **e** Results of western blot analysis indicated that the miR-617 inhibitor rescued the si-circSIPA1L1-3-mediated downregulation of RUNX2, ALP, OSX, and Smad3. *P < 0.05, **P < 0.01. **f** Results of western blotting were analyzed with ImageJ software, and data were presented as ratio of target protein to GAPDH in the form of grayscale value. (*P < 0.05, **P < 0.01)

To test the interaction between miR-617 and Smad3, pciCHECK2-Smad3 and psiCHECK2-mut-Smad3 were constructed. Dual-luciferase reporter assay uncovered decreased luciferase activity after co-transfection of miR-617 mimics and pciCHECK2-Smad3, confirming the direct interaction between miR-617 and Smad3 (Fig. 5d). Interestingly, the Smad3 level was positively regulated by circSIPA1L1, but negatively regulated by miR-617. CircSIPA1L1/miR-617 induces osteogenic differentiation of DPSCs by targeting Smad3. Western blot assay showed higher protein levels of Smad3 in the NC group and miR-617 inhibitor than the miR-617 mimic group and miR-617 inhibitor NC group, respectively. Meanwhile, the Si-NC group showed higher protein levels of Smad3 than the Si-circSIPA1L1-3 group and Si-circSIPA1L1-1 group, respectively (Fig. 6a–c). Immunofluorescence assay revealed a similar result (Fig. 6d).

### MiR-617 reversed the regulatory effect of circSIPA1L1 on DPSC osteogenesis

Rescue experiments were conducted to clarify the involvement of miR-617/Smad3 in circSIPA1L1-mediated osteogenesis. Western blot results showed that downregulated RUNX2, ALP, OSX, and Smad3 in osteogenic DPSCs with circSIPA1L1 knockdown were partially reversed by co-silence of miR-617 (Fig. 6e, f).

## Discussion

In recent years, critical functions of circRNAs in human diseases have been highlighted, which may provide a theoretical basis for developing novel treatments [19, 27, 28]. Serving as ceRNAs, circRNAs can sponge miRNAs, proteins, and trans-acting elements, thus influencing gene transcription, expressions, and functions. In this paper, we focused on the role of circSIPA1L1 in the bone regeneration and its potential mechanism.

Owing to the multilineage differentiation potential, DPSCs are considered as candidates in bone regeneration. Our finding showed that circSIPA1L1 was dynamically upregulated during DPSC osteogenesis, while miR-617 showed the opposite trend. CircSIPA1L1 was unable to influence the proliferative potential of osteogenic DPSCs. However, it indeed stimulated osteogenesis of DPSCs as ALP and ARS staining indicated. As an early marker of calcification, ALP is linked to osteoblast activity and osteogenesis specificity [29]. Its expression and activity were markedly enhanced in the early stage of mineralization. RUNX2 is a transcriptional regulator responsible for early-stage osteogenesis, which directly affects gene expressions associated with intracranial secretion and bone tissue enrichment. Studies have shown that RUNX2 knockout mice performed a complete lack of the bone formation [30]. OSX is a critical downstream gene of RUNX2 that is involved in the bone formation and osteoblast differentiation. It is reported that MSCs isolated from OSX-deficient mice cannot be differentiated into osteoblasts [31]. Here, osteogenesis markers (e.g., ALP, RUNX2, OSX) were found to be positively regulated by circSIPA1L1, further confirming our findings. In addition, a subcutaneous transplantation model in nude mice by cell scaffold material was established. Histological examination results were consistent with the in vitro conclusions. Taken together, these findings indicated that circSIPA1L1-stimulated DPSC osteogenesis.

Recently, ceRNA hypothesis proposed a vital regulatory loop, that is, circRNA-miRNA-mRNA axis [32]. For instance, circNRIP1 aggravates gastric cancer progression by sponging microRNA-149-5p via the AKT1/mTOR pathway [33]. In addition, circHIPK3 promotes the proliferative and differentiation potentials of chicken myoblasts by sponging miR-30a-3p [34]. Vital functions of miRNAs in stem cell regulation have been well concerned. Many miRNAs have been identified to participate in osteoblast differentiation processes [35]. For example, miR-21, miR-26a, and miR-196 are involved in MSC osteogenesis [36–38]. To explore the potential contributing mechanisms of circSIPA1L1 in DPSC osteogenesis, bioinformatics analysis was conducted to seek potential targets binding circSIPA1L1. Our results demonstrated that miR-617 was the target gene binding circSIPA1L1 through dual-luciferase reporter assay, which negatively mediated DPSC osteogenesis. Notably, miR-617 was capable of abolishing the regulatory effect of circSIPA1L1 on DPSC osteogenesis. In the cytoplasm, ceRNAs can affect mRNA stability and translational regulation under the circumstances that two interacted genes should be colocalized [39, 40]. FISH results illustrated that circSIPA1L1 and miR-617 were co-localized in the cytoplasm of DPSCs.

In a similar way, Smad3 was discovered to be a downstream gene of miR-617, which is an important component of TGF-β signaling. Smad3 (mothers against decapentaplegic homolog 3) is a crucial regulator of TGF-β/Smads pathway [41]. It is able to mediate the synthesis and degradation of the extracellular matrix, as well as cell phenotypes [42, 43]. After TGF-β and RUNX2 induction, Smads are activated and accumulated to contribute to skeleton formation [44]. It is reported that miR-708 can effectively abolish the inhibitory effect of Dex on osteoblast differentiation by upregulating Smad3 [45]. In this paper, Smad3 was proven to be the downstream gene binding miR-617. Its level was positively regulated by circSIPA1L1 and negatively regulated by miR-617. Therefore, we hypothesized a circSIPA1L1/miR-617/Smad3 axis responsible for mediating DPSC osteogenesis. Overexpression of inflammation-induced miR-223-3p triggers odontoblast differentiation of DPSCs by targeting Smad3 [46]. The synergistic activity of Smads following Runx2 activation is of significance in the bone formation. The Smad pathway mediates the differentiation of mesenchymal progenitors through converging RUNX2 [47]. Taken together, we believed that the circSIPA1L1/miR-617/Smad3 axis stimulated DPSC osteogenesis.

## Conclusion

CircSIPA1L1 is dynamically upregulated under mineralization-inducing conditions. CircSIPA1L1/miR-617/Smad3 axis is responsible for stimulating DPSC osteogenesis, which can be utilized as bone regeneration targets.

## Supplementary information

**Additional file 1: Figure S1.** Phenotype identification of DPSCs. **A**. Morphology of primary generation DPSCs **B.** Flow cytometry showed that DPSCs were negative for hematopoietic markers of CD34 and CD45. **C.** Flow cytometry demonstrated that DPSCs presented positive for CD29, CD73, CD90 and CD105. **D.** Trilineage differentiation (adipogenic, osteogenic and chondrogenic differentiations) of DPSCs analyzed by Oil red O staining, Alizarin red S staining and Alcian blue staining respectively. Immunofluorescence assay revealed that cultured DPSCs were positive for STRO-1. Scale bar = 100 μm. **Figure S2.** CircSIPA1L1 have no effect on DPSCs proliferation. **A.** Cell cycle phases in different group for proliferation index (PI=G2M+S) by flow cytometry analysis. **B-D.** EdU assay showed no significant difference in EdU-positive cell ratio between NC group and circSIPA1L1 group or between the Si-NC, Si-circSIPA1L1-1 and Si-circSIPA1L1-3 groups (N.S., *P > 0.05*). **E, F.** The influences of circSIPA1L1 on the cell proliferation capability was detected at 450 nm with CCK-8. CCK-8 assay showed no significant difference in cell proliferation between NC group and circSIPA1L1 group or between the Si-NC, Si-circSIPA1L1-1 and Si-circSIPA1L1-3 groups from 0 to 9 days (*P*>.05). CCK-8, cell counting kit-8; DPSCs, dental pulp stem cells; EdU, 5-ethynyl-20-deoxyuridine assay; PI, propidium iodide. N.S: *P* > 0.05.

**Additional file 2.**

## Data Availability

The datasets used and analyzed during the current study are available from the corresponding author on reasonable request.

## Abbreviations

CircSIPA1L1: Circular RNA SIPA1L1
DPSCs: Dental pulp stem cells
miRNA: MicroRNA
ARS: Alizarin red staining
RT-PCR: Reverse transcription polymerase chain reaction
ALP: Alkaline phosphatase
RUNX2: Runt-related transcription factor 2
MSCs: Mesenchymal stem cells
ceRNA: Competing endogenous RNA
3′UTR: 3′ Untranslated region
NC: Negative control
