# Supplementary material for: Circular RNA SIPA1L1 promotes osteogenesis via regulating the miR-617/Smad3 axis in dental pulp stem cells
Source: Stem Cell Res Ther. 2020 Aug 24;11:364. doi: 10.1186/s13287-020-01877-3 (PMC7444204; doi:10.1186/s13287-020-01877-3)
Supplement: Supplementary file 1 — Additional file 1: Figure S1. Phenotype identification of DPSCs. A. Morphology of primary generation DPSCs B. Flow cytometry showed that DPSCs were negative for hematopoietic markers of CD34 and CD45. C. Flow cytometry demonstrated that DPSCs presented positive for CD29, CD73, CD90 and CD105. D. Trilineage differentiation (adipogenic, osteogenic and chondrogenic differentiations) of DPSCs analyzed by Oil red O staining, Alizarin red S staining and Alcian blue staining respectively. Immunofluorescence assay revealed that cultured DPSCs were positive for STRO-1. Scale bar = 100 μm. Figure S2. CircSIPA1L1 have no effect on DPSCs proliferation. A. Cell cycle phases in different group for proliferation index (PI=G2M+S) by flow cytometry analysis. B-D. EdU assay showed no significant difference in EdU-positive cell ratio between NC group and circSIPA1L1 group or between the Si-NC, Si-circSIPA1L1-1 and Si-circSIPA1L1-3 groups (N.S., P > 0.05). E, F. The influences of circSIPA1L1 on the cell proliferation capability was detected at 450 nm with CCK-8. CCK-8 assay showed no significant difference in cell proliferation between NC group and circSIPA1L1 group or between the Si-NC, Si-circSIPA1L1-1 and Si-circSIPA1L1-3 groups from 0 to 9 days (P>.05). CCK-8, cell counting kit-8; DPSCs, dental pulp stem cells; EdU, 5-ethynyl-20-deoxyuridine assay; PI, propidium iodide. N.S: P > 0.05. [file 13287_2020_1877_MOESM1_ESM.docx]

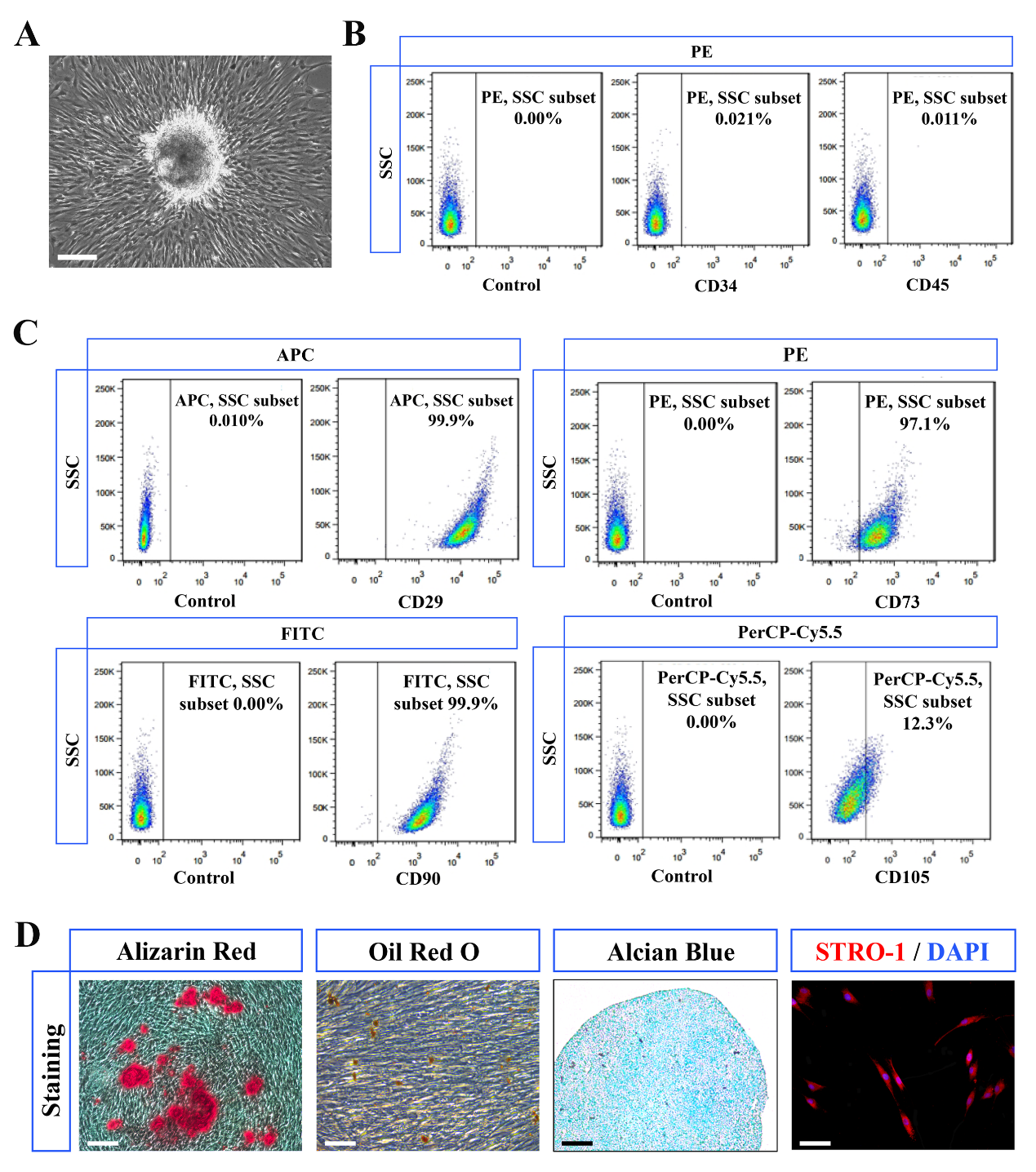


**Figure S1. Phenotype identification of DPSCs. A**. Morphology of primary generation DPSCs **B.** Flow cytometry showed that DPSCs were negative for hematopoietic markers of CD34 and CD45. **C.** Flow cytometry demonstrated that DPSCs presented positive for CD29, CD73, CD90 and CD105. **D.** Trilineage differentiation (adipogenic, osteogenic and chondrogenic differentiations) of DPSCs analyzed by Oil red O staining, Alizarin red S staining and Alcian blue staining respectively. Immunofluorescence assay revealed that cultured DPSCs were positive for STRO-1. Scale bar = 100 μm.


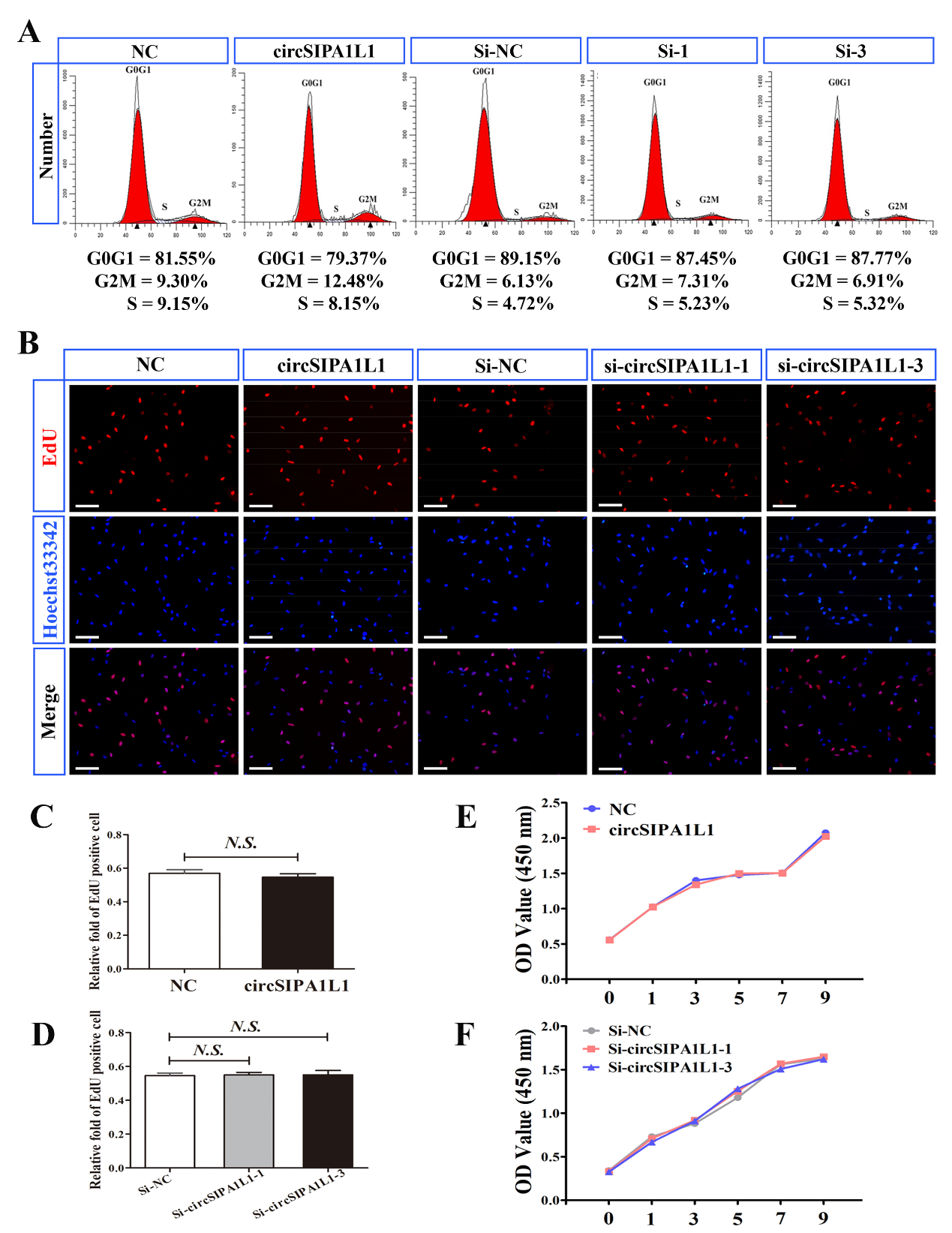


**Figure S2. CircSIPA1L1 have no effect on DPSCs proliferation.** **A.** Cell cycle phases in different group for proliferation index (PI=G2M+S) by flow cytometry analysis. **B-D.** EdU assay showed no significant difference in EdU‐positive cell ratio between NC group and circSIPA1L1 group or between the Si-NC, Si-circSIPA1L1-1 and Si-circSIPA1L1-3 groups (N.S., *P > 0.05*). **E, F.** The influences of circSIPA1L1 on the cell proliferation capability was detected at 450 nm with CCK-8. CCK‐8 assay showed no significant difference in cell proliferation between NC group and circSIPA1L1 group or between the Si-NC, Si-circSIPA1L1-1 and Si-circSIPA1L1-3 groups from 0 to 9 days (P>.05). CCK‐8, cell counting kit‐8; DPSCs, dental pulp stem cells; EdU, 5‐ethynyl‐20‐deoxyuridine assay; PI, propidium iodide. N.S: *P* > 0.05.
